# Multi-site comparison of parametric T1 and T2 mapping: healthy travelling volunteers in the Berlin research network for cardiovascular magnetic resonance (BER-CMR)

**DOI:** 10.1186/s12968-023-00954-9

**Published:** 2023-08-14

**Authors:** Jan Gröschel, Ralf-Felix Trauzeddel, Maximilian Müller, Florian von Knobelsdorff-Brenkenhoff, Darian Viezzer, Thomas Hadler, Edyta Blaszczyk, Elias Daud, Jeanette Schulz-Menger

**Affiliations:** 1 Charité - Universitätsmedizin Berlin, corporate member of Freie Universität Berlin and Humboldt-Universität Zu Berlin, ECRC Experimental and Clinical Research Center, Lindenberger Weg 80, 13125 Berlin, Germany; 2 Working Group On Cardiovascular Magnetic Resonance, Experimental and Clinical Research Center, a joint cooperation between Charité Medical Faculty and the Max-Delbrück Center for Molecular Medicine, Lindenberger Weg 80, 13125 Berlin, Germany; 3 DZHK (German Centre for Cardiovascular Research), Partner Site Berlin, Berlin, Germany; 4 Department of Anaesthesiology and Intensive Care Medicine, Campus Benjamin Franklin, Charité, Universitätsmedizin Berlin, corporate member of Freie Universität Berlin Und Humboldt-Universität Zu Berlin, Berlin, Germany; 5 KIZ, Kardiologie im Zentrum, Eisenmannstr. 4, 80331 Munich, Deutschland; 6 The Cardiology Department, Galilee Medical Center, Azrieli Faculty of Medicine Bar-Ilan University, Nahariya, Safed, Israel; 7 Department of Cardiology and Nephrology, HELIOS Hospital Berlin-Buch, Berlin, Germany

**Keywords:** Cardiovascular magnetic resonance, Parametric mapping, Validation, Reproducibility, Post-processing

## Abstract

**Background:**

Parametric mapping sequences in cardiovascular magnetic resonance (CMR) allow for non-invasive myocardial tissue characterization. However quantitative myocardial mapping is still limited by the need for local reference values. Confounders, such as field strength, vendors and sequences, make intersite comparisons challenging. This exploratory study aims to assess whether multi-site studies that control confounding factors provide first insights whether parametric mapping values are within pre-defined tolerance ranges across scanners and sites.

**Methods:**

A cohort of 20 healthy travelling volunteers was prospectively scanned at three sites with a 3 T scanner from the same vendor using the same scanning protocol and acquisition scheme. A Modified Look-Locker inversion recovery sequence (MOLLI) for T1 and a fast low-angle shot sequence (FLASH) for T2 were used. At one site a scan-rescan was performed to assess the intra-scanner reproducibility. All acquired T1- and T2-mappings were analyzed in a core laboratory using the same post-processing approach and software.

**Results:**

After exclusion of one volunteer due to an accidentally diagnosed cardiac disease, T1- and T2-maps of 19 volunteers showed no significant differences between the 3 T sites (mean ± SD [95% confidence interval] for global T1 in ms: site I: 1207 ± 32 [1192–1222]; site II: 1207 ± 40 [1184–1225]; site III: 1219 ± 26 [1207–1232]; *p* = 0.067; for global T2 in ms: site I: 40 ± 2 [39–41]; site II: 40 ± 1 [39–41]; site III 39 ± 2 [39–41]; *p* = 0.543).

**Conclusion:**

Parametric mapping results displayed initial hints at a sufficient similarity between sites when confounders, such as field strength, vendor diversity, acquisition schemes and post-processing analysis are harmonized. This finding needs to be confirmed in a powered clinical trial.

*Trial registration* ISRCTN14627679 (retrospectively registered)

**Supplementary Information:**

The online version contains supplementary material available at 10.1186/s12968-023-00954-9.

## Background

Non-invasive quantitative myocardial tissue characterization based on parametric T1- and T2-mapping has entered clinical application several years ago and has proceeded to be one of the main techniques applied in contemporary cardiovascular magnetic resonance (CMR) imaging [1]. In order to reach this prominent position, several studies laid the foundation, reporting results regarding validation, accuracy, precision and value ranges for healthy myocardium [2–7]. Based on these findings, other publications presented insights into patient centered outcomes and the value of parametric tissue differentiation regarding diagnosis as well as treatment [8–10]. Additionally the application of parametric mapping added valuable insights into understanding the effect aging has on the myocardium [6, 11]. In spite of these major diagnostic advantages and research opportunities, parametric mapping suffers from the lack of generalizable results between scanners and sites [7, 12]. Given its intrinsic magnetic depending properties, values in healthy and diseased hearts vary based on a myriad of factors [12, 13]. These factors can be divided into technical ones such as field strength, scanner version and vendor diversity, sequence design, body coils used, as well as physiological ones such as gender, age, body temperature and lastly methodological ones such as post-processing approach and software [7, 12, 14]. On the other hand, some factors like small variations in spatial resolution remain without effect on the native T1 relaxation times [15]. The interaction of these factors leads to a complex interdependence which has impeded multicenter studies. Previous approaches to overcome these issues have focused on post-processing steps to account for inter-scanner differences. A popular one being the so-called Z-score where quantitative T1- and T2-values are converted into unitless relative numbers [16]. This approach omitted differences between field strengths, vendors and sequence design. However, there is still a lack of knowledge whether in standardized conditions, where the majority of the technical factors are controlled, equivalent results can be achieved in vivo. This study aims to provide insights and data whether multi-site studies that account and control for confounding factors might be able to provide equivalent parametric mapping values over scanner and sites.

## Methods

### Study cohort

A cohort of N = 20 healthy volunteers was prospectively recruited and screened for eligibility. Participants were eligible to participate in the study in the absence of any cardiovascular, pulmonary, endocrine, or renal conditions and gave written informed consent and were over the age of 18 years. Exclusion criteria were any contraindications for CMR, pregnancy, breastfeeding or claustrophobia. Ethical approval was obtained from the local ethics committee of Charité Medical University Berlin (approval number EA1/183/19). The study was retrospectively registered (ISRCTN14627679).

### Study sites

**Fig. 1:**
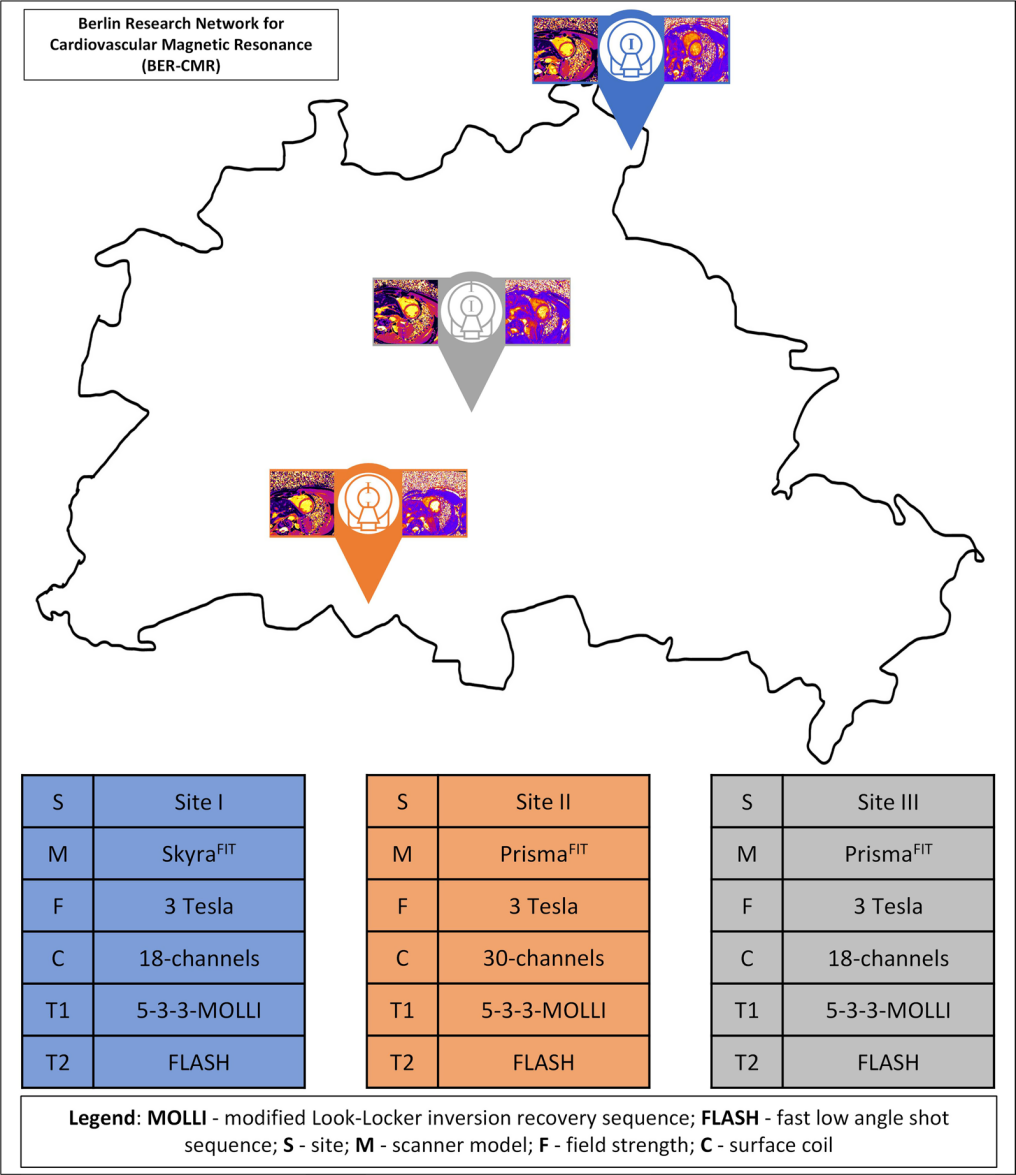
Illustration representing the Berlin research network for cardiovascular magnetic resonance (BER-CMR). Each colored pin represents one scan site. Corresponding information regarding each site, which includes the scanner type, field strength, coil and T1 as well as T2 sequence used, are presented

All participants underwent a CMR scan at each of the following sites of the Berlin Research Network for CMR (BER-CMR): site I with 3 T scanner (Skyra^FIT^), sites II and III with 3 T scanners (Prisma^FIT^) (all Siemens Healthineers, Erlangen, Germany). Scanner at site II is used for clinical scans, the other scan sites are research scanners only. Sites II and III were trained before the start of the study. During the study scans were monitored by vendor provided software (expert-I, Siemens Healthineers, Erlangen Germany). At scanner sites I and III an 18-channel body surface coil was used and at scanner site II a 30-channel body surface coil was used (Fig. 1).

### Study protocol

After acquiring localizers for positioning, cine imaging for assessment of cardiac function was carried out by balanced steady-state free-precession (bSSFP) sequences. A full short axis (SAX) stack covering the entire left ventricle (LV) and four long axes including a 4-chamber view (cv), 3-cv and 2-cv as well as a right ventricular (RV) view, were acquired. Parametric T1 -and T2-mapping was acquired in the three SAX slices: basal, midventricular and apical, based on the 3-out-of-5 method [17]. T1-mapping was based on a motion corrected Modified Look-Locker inversion recovery sequence (MOLLI) in a 5-3-3 scheme. This acquisition scheme is based on the initially suggested MOLLI sequence by Messroghli et al. with a 3-3-5 pattern [3, 18]. In the 5-3-3 pattern, applied in the current study, 5 images are acquired after an initial inversion 180° pulse, followed by the 3 heart beats without acquisition. After another inversion pulse, 3 more images are acquired [4, 19]. T2-mapping was based on a motion corrected fast low-angle shot (FLASH) sequence. This technique acquires 3 images, each with a varying T2 preparation time before each image [20]. In the current acquisition scheme preparation durations were 0-30-55, as a slight modification of previous works [21, 22]. Sequence details are provided in Table 1. To ensure that each volunteer scan at the different sites was carried out with the same adjustable image parameters, such as distance factor and field of view, at the first scan site, these settings were noted and applied to the consecutive scans. At study site I the volunteers were rescanned after exiting the scanner and waiting for 15 min.

**Table 1:** Scanner and sequence parameters for T1 and T2 mapping acquisitions

Parameter	Site I	Site II	Site III
Field strength (T)	3	3	3
Vendor	Siemens	Siemens	Siemens
Model	Skyra^FIT^	Prisma^FIT^	Prisma^FIT^
RF-coil	18-channel	32-channel	18-channel
Bore size (cm)	70	60	60
Bore size (cm)	70	60	60
T1 scan parameters			
Sequence	5-3-3 MOLLI	5-3-3 MOLLI	5-3-3 MOLLI
Slice thickness (mm)	6.0	6.0	6.0
Voxel size (mm × mm)	1.4 × 1.4	1.4 × 1.4	1.4 × 1.4
Field of view (mm)	360	360	360
Echo time (ms)	1.12	1.12	1.12
Repetition time (ms)	3.9	3.9	3.9
Flip angle (o)	35	35	35
Bandwith (Hz/Px)	1085	1085	1085
Acceleration factor	2	2	2
T2 scan parameters			
Sequence	FLASH	FLASH	FLASH
Slice thickness (mm)	6.0	6.0	6.0
Voxel size (mm × mm)	1.9 × 1.9	1.9 × 1.9	1.9 × 1.9
Field of view (mm)	360	360	360
Echo time (ms)	1.32	1.32	1.32
Repetition time (ms)	3.6	3.6	3.6
Flip angle (degrees)	12	12	12
Bandwidth (Hz/Px)	1184	1184	1184
Acceleration factor	2	2	2

Modified Look-Locker inversion recovery sequence (MOLLI); fast low-angle shot (FLASH); steady-state free-precession (SSFP)

### CMR analysis

All images were analyzed with dedicated commercially available software (CVI42, Version 5.13.7, Calgary, Alberta, Canada) by two readers with 8 (R.F.T.) and 3 years (J.G.) experience in CMR. Cardiac function and mass were evaluated as per current recommendations in SAX cine images with delineation of papillary muscles [23, 24]. Mapping analysis was done in all three SAX slices. Endocardial and epicardial contours were drawn in the grey scale images avoiding contouring the blood pool, the epicardium, or the RV. An offset of 5% that shifts the contours towards the myocardial center was used within the analysis software in order to securely segment myocardial tissue only. After demarcation of the long axis extent of the LV, bull’s eye plots according to the American Heart Association model with 16-segments were constructed. Global and slice-based values for basal, midventricular and apical segments were analyzed. Additionally, septal values in SAX for basal and midventricular slices were provided. Each slice, including source images, was reviewed carefully for artifacts and proper motion correction. Segments with artefacts were excluded from the final analysis. Intra- and interobservers differences were assessed based on 12 different, randomly selected scans, 3 from each site.

### Statistical analysis

Given normal distribution, as assessed by the Shapiro Wilk test, all continuous variables are given as mean ± standard deviation (SD) with the 95%-confidence interval and, where appropriate, as percentage. All categorical variables are given as total and percent. Comparisons for global, basal, midventricular, apical as well as basal and midventricular septal means for T1 and T2 were carried out for sites I, II and III. In addition, AHA segment means were compared. As a global test a repeated measures ANOVA was applied. In cases in which the significance level set at < 0.05 was reached, pairwise comparisons were carried out between the sites with a Bonferroni correction. The 95%-confidence intervals of the differences between sites I, II and III were calculated and compared to previously defined 95%-tolerance intervals (for T1 mapping ± 24.5 ms and for T2 mapping ± 3.2 ms) to assess for equivalence [25]. Equivalence was established if the confidence intervals of the difference between the two sites was within the tolerance intervals [25]. Intra- and inter-reader comparisons based on 9 cases as well as the scan-rescans at site I were compared by Bland–Altman plots with 95%-limits of agreement. To provide a percentual number of slices meeting the quality standard we divided the number of analyzed segments by the maximum possible number of segments. The maximum number of available segments were 16 per patient. Statistical analysis was conducted with dedicated software (SPSS Statistics Version 27.0.0, IBM, Armonk, New York, USA).

## Results

### Study cohort

Of the 20 volunteers one had to be excluded from the final analysis as a cardiovascular comorbidity was diagnosed shortly after the scans. From the remaining 19 volunteers (8 females/ 11 males; (mean ± SD) age 26.1 ± 6 years; weight 70.2 ± 11.4 kg; height 1.8 ± 0.1 m; body mass index 21.7 ± 2.4 kg/m^2^; body surface area 1.9 ± 0.2 m^2^) all underwent scans at site II and 18 at sites I and III. LV and RV function parameters, provided as total and indexed concerning body surface area and height where appropriate, were as follows: LV end-diastolic volume: 181 ± 42 ml/96 ± 17 ml/m^2^/100 ± 20 ml/m; LV stroke volume: 113 ± 27 ml/60 ± 17 ml/m^2^; LV ejection fraction: 62 ± 3%; RV end-diastolic volume: 204 ± 51 ml/108 ± 21 ml/m^2^; RV stroke volume: 107 ± 27 ml/56 ± 11 ml/m^2^; RV ejection fraction: 52 ± 4%. Further general characteristics for the travelling volunteers at each site are presented in Table 2.

**Table 2:** Characteristics of the travelling volunteers and the healthy cohort

Parameter	Site I	Site II	Site III
N =	18	19	18
Female/Male	8/10	8/11	7/11
Age (years)	26.3 ± 6.1	26.1 ± 6	26.3 ± 6.2
Height (m)	1.8 ± 0.1	1.8 ± 0.1	1.8 ± 0.1
Weight (kg)	69.3 ± 11.1	70.2 ± 11.4	70.1 ± 11.7
Body mass index (kg/m^2^)	21.5 ± 2.4	21.7 ± 2.4	21.5 ± 2.4
Body surface area (m^2^)	1.9 ± 0.2	1.9 ± 0.2	1.9 ± 0.2
Heart rate	68.6 ± 12.9	63.8 ± 10.1	69.1 ± 9
Systolic blood pressure (mmHg)	123.2 ± 12.4	119.5 ± 7.4	123.1 ± 9.7
Diastolic blood pressure (mmHg)	69.7 ± 14.4	68.7 ± 11.8	77.5 ± 9.5

Data represented as mean and standard deviation or absolute numbers

### CMR results—quality survey

The highest rate of analyzable segments for T1-mapping was noted at site II with a total of 266/304 segments (88%) used for final analysis after exclusion of artefacts. Sites I (245/288 (85%) showed a similar percentage, with the lowest one at site III (231/288 (80%). Higher overall rates of analyzable segments were found for T2-mapping [site I 288/288 (100%); site II 290/304 (95%); site III 264/272 (97%)].

Of the total of 138 encountered artefacts, 124 (90%) were susceptibility artefacts. The remaining artefacts were due to mispositioning of a slice (basal slice placed toward midventricular region) 12/138 (9%) and 2 [2/138 (1%)] due to motion artefact/cardiac ghosting. Most artefacts were located in the midventricular slices [58/138 (42%)] followed by basal [40/138 (29%)] and apical locations [40/138 (29%)]. Segmental analysis revealed the majority of artefacts being in AHA segment 11 [29/138 (21%)] trailed by segment 16 [24/138 (17%)] and 5 [15/138 (11%)]. Other segments in order: 1 [2/138 (1%)], 2 [2/138 (1%)], 3 (5/138 (4%)], 4 [12/138 (9%)], 6 [4/138 (3%)], 7 [0/138 (0%)], 8 [0/138 (0%)], 9 [4/138 (3%)], 10 [14/138 (10%)], 12 [11/138 (8%)], 13 [2/138 (1%)], 14 [1/138 (1%)], 15 [13/138 (9%)]. The overall rates of slice and segmental involvement were similar if divided by sites. At site I 17/43 (40%) artefacts were in midventricular segments with segment 11 most often involved [8/43 (19%)]. At this site only susceptibility artefacts were encountered. Site II had 17/38 (45%) artefacts in midventricular slices with segment 11 most commonly involved [11/38 (29%)]. Majority [30/38 (79%)] were susceptibility artefacts. Remaining artefacts were due to mispositioned slice [6/38 (16%)] and 2/38 (5%) due to motion artefacts. Overall site III had the most artefacts with 24/57 (42%) in the midventricular slice with segment 11 being most commonly affected [13/57 (23%)]. Susceptibility artefacts accounted for most artefacts [51/57 (89%)] with the other 6 due to a misplaced slice [6/57 (11%)].

### CMR results—travelling volunteers

**Fig. 2:**
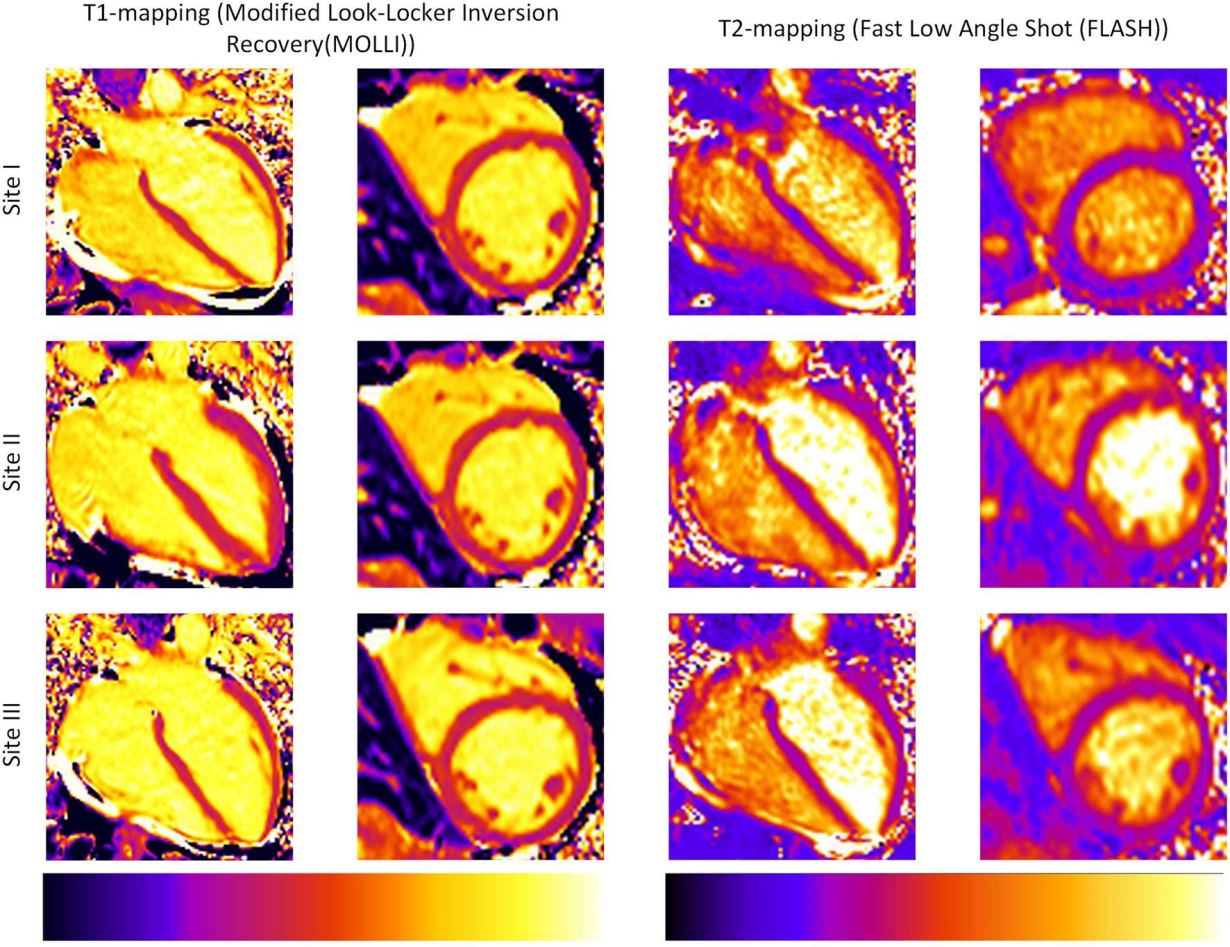
T1- and T2-Mapping acquisitions from one healthy volunteer scanned at all three sites. For each site the mapping acquisitions for T1 and T2 are shown in a four-chamber view (left column) and a midventricular slice (right column). For all acquisitions the same lookup-table was used (shown on the bottom for T1 and T2)

**Fig. 3:**
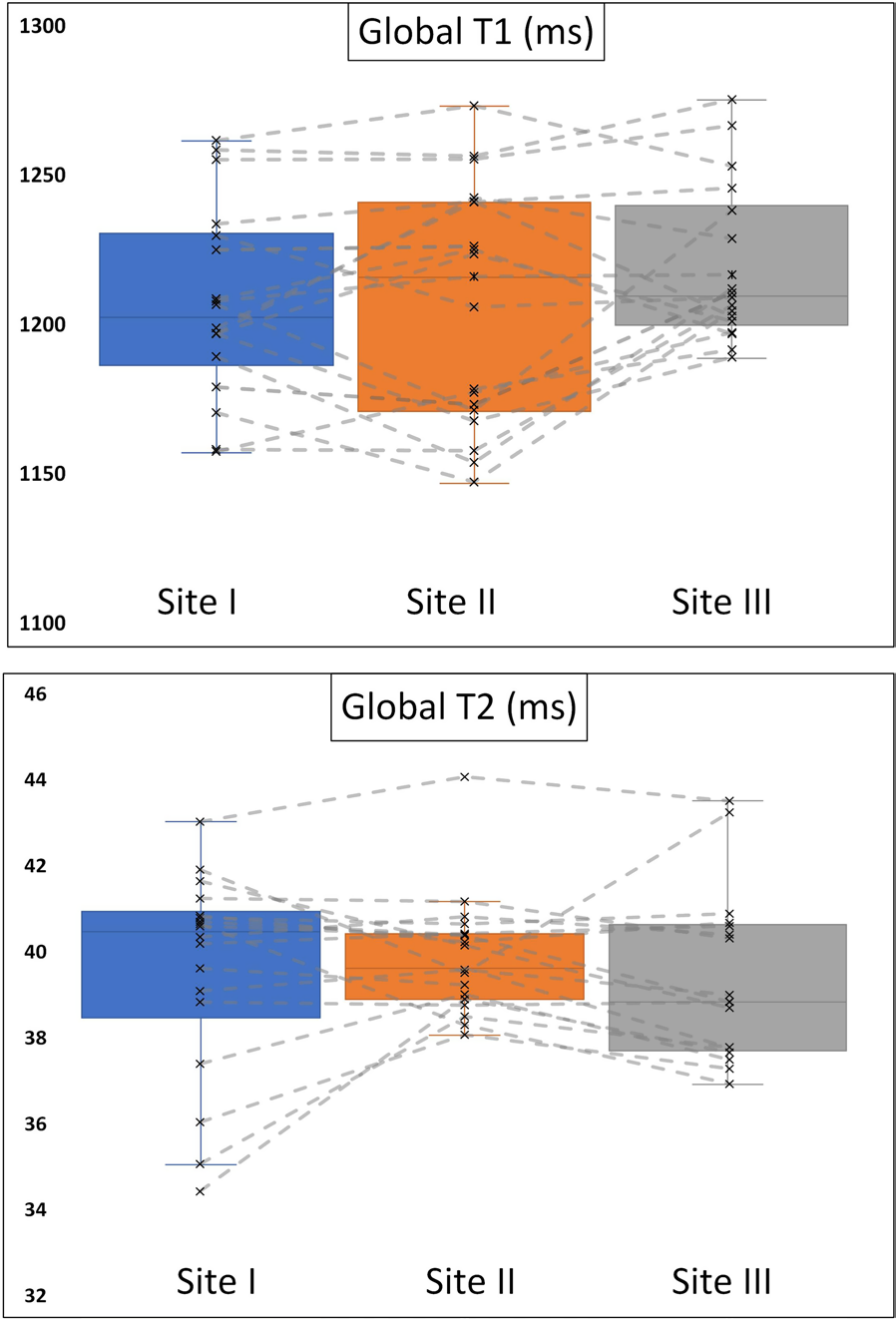
Boxplots for T1- and T2-values for travelling volunteers across the three participating sites. Boxplots representing the median (solid inside the box), interquartile range (box) and 1.5*interquartile range (whiskers) for T1- and T2- mapping at each site (site I blue, site II orange, site III grey). Every value below or above 1.5*interquartile range is marked as an outlier. Grey lines connect each individual travelling volunteer at each scan site

**Fig. 4:**
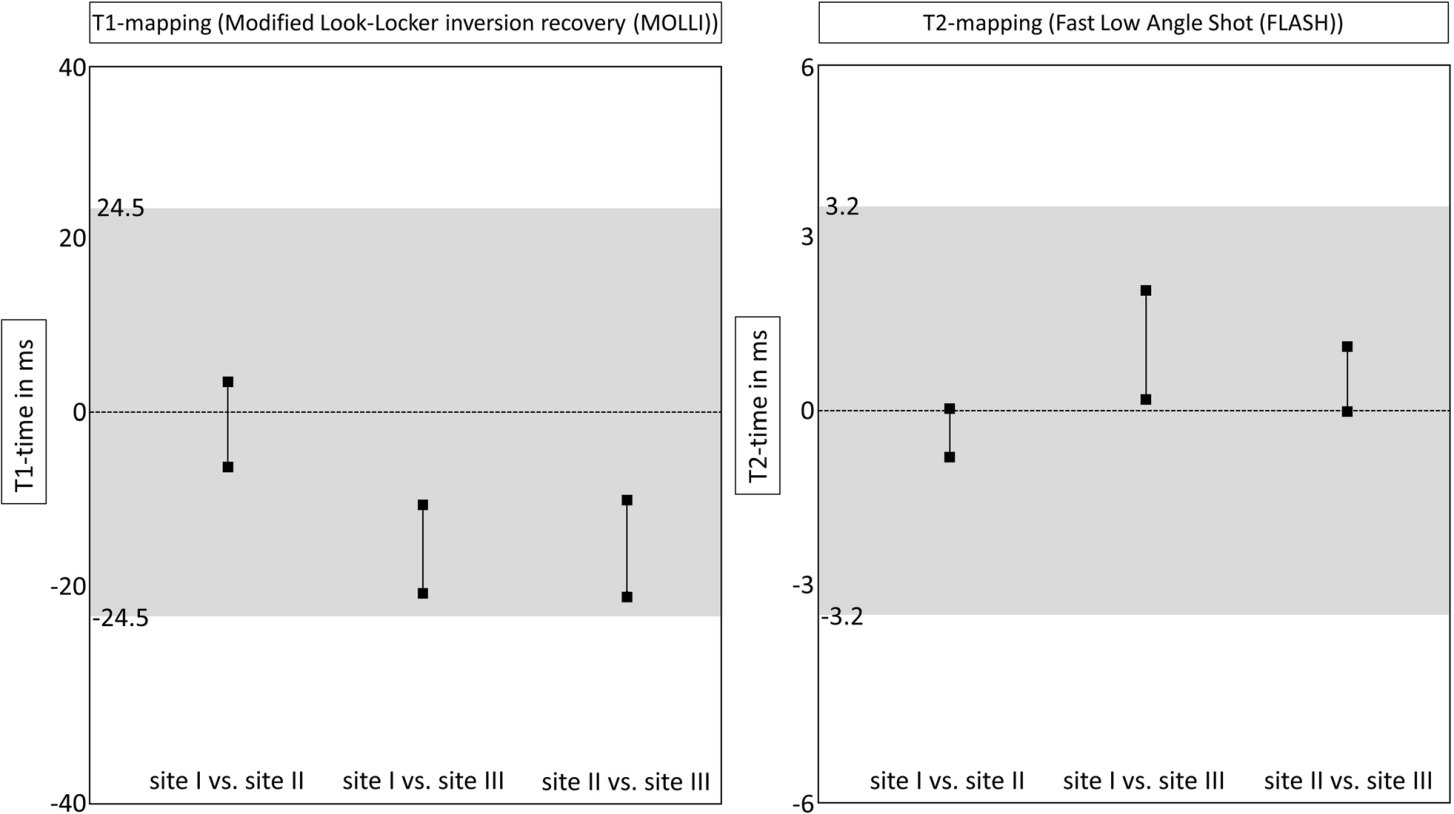
Equivalence testing between sites I, II and III. Equivalence testing for T1- and T2-mapping for the three sites (I, II and III). Equivalence is shown if the 95-% confidence interval of the difference between sites (shown by the black lines with squares marking upper and lower limit) is within the pre-defined equivalence limits (interval marked by grey square). Equivalence limits derived from Zange et al. [25]

**Fig. 5:**
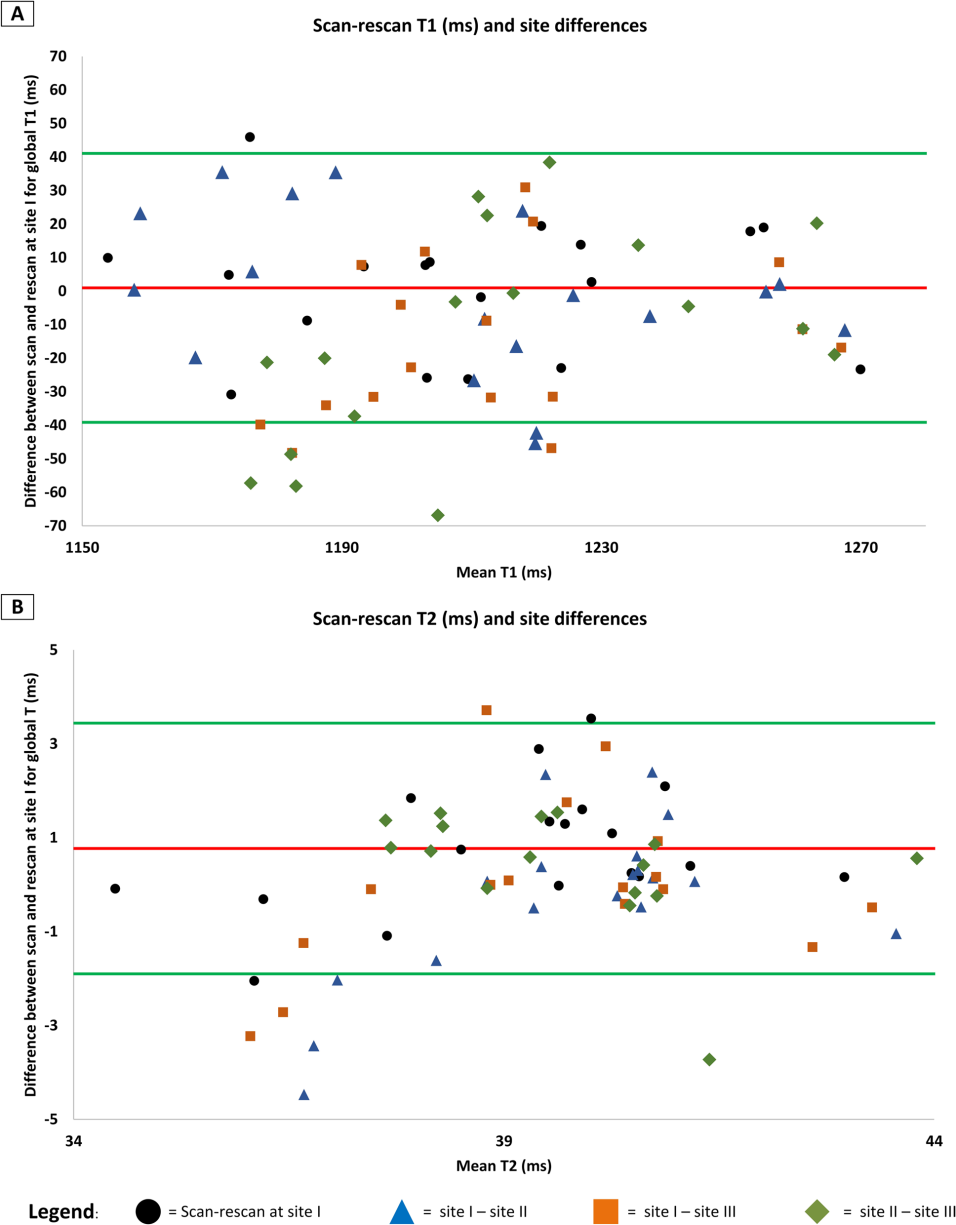
Scan-rescan and inter-site comparisons by Bland–Altman plots. Depicted are scan-rescan comparisons for T1- and T2-mapping (Panel **A** and **B**, respectively) and site differences. Scan-rescan comparisons were carried out at site I with the volunteers exiting the scanner in between scans for 15 min. Red line indicates the mean difference and the green lines indicate the upper and lower limits of agreement. Inter-site differences are marked by the corresponding symbols (black circle = scan-rescan at site I; blue triangle = site I- site II; orange rectangle = site I-site III; green diamond = site II- site III)

Figure 2 provides exemplary mapping acquisitions from one volunteer at all three sites. Global T1 and T2 values showed no significant differences between sites I, II and III (mean ± SD [95% confidence interval] for global T1 in ms site I: 1207 ± 32 [1192–1222]; site II: 1207 ± 40 [1184–1225]; site III: 1219 ± 26 [1207–1232]; *p* = 0.067; for global T2 in ms site I: 40 ± 2 [39–41]; site II: 40 ± 1 [39–41]; site III 39 ± 2 [39–41]]; *p* = 0.543) (Fig. 3). Slice based comparisons for basal, midventricular and apical slices showed no significant differences between the sites except for T1 in midventricular slices (p = 0.028). Pairwise comparisons for midventricular means revealed only significant differences between sites I and III (p = 0.029) (Table 3). On the other hand, septal segments in basal and midventricular slices for sites I, II and III revealed no significant differences for T1 and T2 (basal septal T1 in ms site I: 1227 ± 36 [1211–1244]; site II: 1227 ± 31 [1213–1241]; site III: 1240 ± 36 [1223–1257]; *p* = 0.267; midventricular septal T1 in ms site I: 1222 ± 39 [1206–1238]; site II: 1224 ± 37 [1208–1241]; site III: 1232 ± 29 [1218–1245]; *p* = 0.202; for basal septal T2 in ms site I: 40 ± 3 [39–41]; site II: 40 ± 1 [39–41]; site III 40 ± 2 [39–41]; *p* = 0.815; for midventricular septal T2 in ms site I: 41 ± 3 [40–43]; site II: 41 ± 2 [40–42]; site III 41 ± 3 [40–42]; *p* = 0.898) (Table 3). Segmental comparisons provided significant differences between the sites for segment 12 (p = 0.04), with a pairwise test tracing the significant difference between sites I and III (p = 0.003). The 95-% confidence interval of the difference between sites I, II and III were inside the pre-defined 95-% tolerance ranges for T1 and T2 (Fig. 4 and Table 4). Scan-rescan analysis revealed narrow limits of agreements as visualized by the Bland-Altmann plots (Fig. 5). Intra- and inter-reader comparisons can be found in Additional File 1.

**Table 3:** T1 and T2 mapping results for the travelling volunteers

Parameter	Site I	Site II	Site III	p value for sites I vs. II vs. III
T1 global (ms)	1207 ± 32 [1192–1222]	1207 ± 40 [1184–1225]	1219 ± 26 [1207–1232]	0.067
T1 basal (ms)	1212 ± 27 [1200–1225]	1210 ± 33 [1194–1226]	1224 ± 27 [1210–1237]	0.178
T1 midventricular (ms)	1205 ± 34 [1189–1220]	1207 ± 40 [1188–1225]	1219 ± 26 [1206–1231]	0.028*
T1 apical (ms)	1203 ± 40 [1184–1221]	1199 ± 51 [1175–1222]	1214 ± 33 [1198–1229]	0.089
T1 basal septum (ms)	1227 ± 36 [1211–1244]	1227 ± 31 [1213–1241]	1240 ± 36 [1223–1257]	0.267
T1 midventricular septum (ms)	1222 ± 39 [1206–1238]	1224 ± 37 [1208–1241]	1232 ± 29 [1218–1245]	0.202
T2 global (ms)	40 ± 2 [39–41]	40 ± 1 [39–41]	39 ± 2 [39–41]	0.543
T2 basal (ms)	39 ± 2 [38–40]	39 ± 1 [39–40]	39 ± 2 [38–40]	0.546
T2 midventricular (ms)	40 ± 2 [39–41]	40 ± 2 [39–41]	40 ± 2 [39–41]	0.954
T2 apical (ms)	40 ± 3 [39–42]	41 ± 2 [40–42]	40 ± 2 [39–41]	0.143
T2 basal septum (ms)	40 ± 3 [39–41]	40 ± 1 [39–41]	40 ± 2 [39–41]	0.815
T2 midventricular septum (ms)	41 ± 3 [40–43]	41 ± 2 [40–42]	41 ± 3 [40–42]	0.898

Bold typed values are significant (*p*<0.05)

Data represented as mean and standard deviation with 95% confidence in square brackets

*pairwise testing with Bonferroni correction revealed statistically significant differences for site I vs. site III (p = 0.029)

**Table 4:** Differences of the T1 and T2 mapping results for the travelling volunteers between sites I, II and III

Parameter	Site I vs. site II	Site I vs. site III	Site II vs. site III
T1 global (ms/%)	1.4 [− 3.2;6.1] / 0.1 [− 0.9; 1]	− 15.8 [− 20.5; -11.5)] / 1.2 [0.2; 2.1]	− 15.7 [− 21.0; − 10.4)]/ 1.1 [− 0.1; 2.3]
T1 basal (ms/%)	− 0.6 [− 8.5; 7.2] / 0.1 [− 1.0; 1.2]	− 15.9 [− 22.9; − 8.9)] / 1 [− 0.1; 2.0]	− 14.3 [− 23.3; − 5.4)] / 1.1 [− 0.4; 2.6]
T1 midventricular (ms/%)	0.1 [− 6.7; 6.9] / 0.3 [− 0.7; 1.3]	− 16.3 [− 22.8; − 9.7] / 1.5[0.5; 2.4]	− 14.8 [− 22.5; − 7.2] /1.1 [0.1; 2.1]
T1 apical (ms/%)	7.2 [− 2.5; 16.9] / − 0.3 [− 1.3; 0.7]	− 14.7 [− 24.4; 5.1)] / 1.1 [0.01; 2.2]	− 19.1 [− 30.7; − 7.4)]/ 1.3 [− 0.2; 2.8]
T2 global (ms/%)	− 0.4 [− 0.7; 0.0] / 0.8 [− 1.3; 2.9]	1.1 [0.2; 2.0] / 0.0 [− 2.3–2.3]	0.6 [− 0.2; 0.9] / − 1.3 [− 2.8; 0.2]
T2 basal (ms/%)	− 0.4 [− 0.9; 0.2] / 0.9 [− 1.1; 3.0]	0.3 [− 0.7; 1.3] / 0.3 [− 2.2; 2.7]	0.2 [− 0.4; 0.7] / − 0.6 [− 2.6; 1.5]
T2 midventricular (ms/%)	0.0 [− 0.6; 0.5] / 0.05 [− 2.3; 2.4]	1.1 [− 0.5; 2.7] / 0.1 [− 2.3; 2.6]	− 0.4 [− 1.3; 0.5] − 0.7 [− 2.8; 1.4]
T2 apical (ms/%)	− 0.9 [− 1.8; 0.0] / 2.1 [− 1.0; 5.3]	2.2 [− 0.1; 4.5] / − 0.8 [− 4.2; 2.7]	1.8 [0.5; 3.2] / − 3.4 [− 5.5; − 1.4]

Data presented for differences between sites as absolute and percent numbers (95% confidence interval in square brackets)

## Discussion

This study aimed at sharing insights into how parametric mapping results can be standardized across different sites in order to lay a foundation for future multicenter studies. If major confounders, which in our analysis included scanner field strength, intra-vendor diversity, sequence parameters, scan parameters and post-processing approach, are controlled and set across all participating sites, parametric T1- and T2-mapping results are equivalent between the different scan sites for the same field strength. However even small differences such as coils and bore size might induce significant different outcomes. Hence, an unsupervised sequence setup omits the potential of having equivalent outcomes. In addition to the mentioned major confounders other controllable and non-controllable parameters, for example internal scanner settings, intrinsic physiological properties of tissues and artefacts, have to be taken into consideration.

### Native T1- and T2-mapping in multi-site studies

Parametric mapping in CMR can potentially be the next step towards a “non-invasive” biopsy for the characterization and detection of cardiac and systemic disorders with cardiac manifestations, even without the need of contrast media administration. One currently remaining drawback is inter-scanner comparability, which becomes relevant especially in the context of multicenter studies or if one individual patient is followed-up at different scan sites [16]. The current recommendations of the Society for Cardiovascular Magnetic Resonance (SCMR) on quantitative T1- and T2-mapping suggest the establishment of individual reference ranges at each site [12]. The size of the normative collective is dependent on the magnitude of changes that are desired to be detected, ranging from 15 to 20 healthy individuals to up to 50 individuals for smaller and more subtle pathologies [12]. At larger facilities, especially those with research output, these goals are both reachable and feasible [11]. Smaller sites, not being engaged in everyday routine or having access to a healthy collective, are potentially not able to provide these values. This in turn, however, reduces the capacity to provide an accurate diagnosis, which is the main goal of this technique. This discrepancy of expectations towards the technique and the usability as well as applicability has only been partially addressed so far. One approach is to standardize the setup at all participating sites including field strength, vendors, sequence design and post-processing methodology. The “International T1 Multicenter cardiovascular magnetic resonance study” compared 102 healthy subjects scanned across four different sites on 1.5 T and 3 T scanners from the same vendor [14]. The authors reported T1 values of 941 ± 58 ms at 1.5 T and 1072 ± 63 ms at 3 T acquired with a 3-3-5 MOLLI scheme and midventricular slice analysis [14].We observed slightly higher values with lower SD. This might be related to scanner platform, the sequences applied and post-processing software as well as age of the cohorts [16, 25]. Taken together, both studies provide evidence that standardization across different sites tackling the major confounders can provide equivalent mapping results for T1. Our study additionally underpins this by scanning the same participants at three different sites. A study by Piechnik et al. includes a brief report of nine volunteers scanned at two participating sites of which two were scanned at a third site as well [26]. Applying a shortened MOLLI version and the same scanner version and field strength at all sites the interscanner results showed very good agreements between sites with a ± 2 SD of the differences between centers for T1 of 19 ms [26]. These differences lie within the ranges we report in this study. It should be of note, that both studies analyzed three slices on average, underlining the importance of post-processing method chosen [26]. There is still debate whether to analyze the global myocardium or specific segments (see further discussion) [14]. Despite finding a significant difference for the midventricular slice between sites I and III, septal values showed no significant differences across the sites. That meets the current consensus which promotes the septal segments to be the most reliable ones [12]. Another post-processing factor that should be taken into account in each study and kept constant is the offset at which the myocardium is analyzed.

There is less evidence regarding T2 across different sites but previous works at different field strength show similar variances in T2 [27, 28]. In comparison to Baeßler et al. our derived T2-values are lower on both field strength [27] which might be related to the vendor diversity.

### Native T1- and T2-mapping at 3 T

Even small variations, such as vendor and acquisition scheme, can lead to differences in parametric mapping values. To provide further context and insight on the variability we will discuss studies with the same vendor and sequence design. As an example, Yamagata et al. carried out a CMR mapping study in a cohort of 51 healthy subjects [29]. Acquisition schemes, vendor and field strength were similar as in our study, only with differences existing in scanner and post-processing approach [29]. Despite this, T1 values (1200.1 ± 30.7 ms) and T2 values (39.5 ± 1.8 ms) in their study were congruent to ours [29]. The Z-score publication regarding quantitative mapping in CMR included 15 healthy volunteers which were scanned with a 5-3-3 MOLLI scheme using the same scanner as site I [16]. T1 Mapping results (mean 1211 ± 44 ms) of this study were close to the ones reported here [16]. The same scanner type with a 30-channel coil was used by Weingärtner et al., providing mean values for the of 1181 ± 47 for the MOLLI sequence ms taken from 20 healthy volunteers [30]. These differences might however be attributable to a different post-processing approach with ROI placement in each segment. Other studies yielded native myocardial T1 values in a similar range to the ones presented in this study (Texeira et al. 1207.9 ± 18.2 [31], Dong et al. 1202 ± 45 [32], Zhao et al. 1247.73 ± 31.86 [33]). In accordance to missing data regarding T2 travelling volunteer studies, normative values for T2 mapping with a 3 T system and the FLASH sequence are sparse. The previously mentioned study by Yamagata et al. used the same sequence at 3 T with mean values of 39.5 ± 1.8 ms, which is congruent to ranges in our sites [29]. In the same article one can find other smaller studies investigating T2 mapping which also yielded similar results [29]. A recent Meta-Analysis compared T2 times across different vendors and acquisition schemes, with a pooled mean of 46 ms at 3 T [34]. Subgroup analysis of the same vendor at 3 T revealed mean T2 values of 44 ms [34]. To summarize the discussion regarding normal values for T1 and T2 mapping at 3 T, one should look at the recently published results of the Hamburg City Health Study [6]. This large data sample study included 1576 patients of which 129 had no evident cardiovascular risk factors [6]. T1 and T2 acquisition schemes were similar to the ones used here and median T1 and T2 values were 1182 ms and 40 ms, respectively [6]. Despite the accumulated evidence, more research is needed regarding standardization (See following paragraphs).

### Multi-site travelling volunteer studies in CMR

Travelling volunteer studies have been carried out for T2*-mapping [35–37]. These studies included intersite comparisons between countries and vendors. The logistics and planning behind such efforts are challenging. Results reported from these studies show excellent agreement between sites for this technique. One drawback of such studies nonetheless is the drop-out rate, which fortunately was minimal in this study in comparison to previous work [38].

### Confounding factors in multi-site CMR studies

Another challenge that quantitative mapping results are facing is the potential dependency on age, gender and other physiologic parameters. The recent results of the Hamburg city health cohort revealed that female volunteers had higher T1, T2 and ECV values in comparison to males [6]. Interestingly the investigators did not find a causal relationship between age and T1 [6]. This is in contrast to other reports which provide data that T1 increases with age [26]. These conflicting results are underlined by a meta-analysis showing a large variation of T1 across studies [39]. A pooled analysis of studies providing reference values, reports native T1 times for the vendor and sequence used in this study to be 972 ± 43 at 1.5 T and 1196 ± 47 ms for 3 T [7]. These are well within the limits provided in our study. Based on these findings universal reference ranges for parametric mapping acquisitions do not seem to be the answer. Another approach in this regard might be the use of standardized acquisition schemes and sequences, carried out on the same platform, same vendor and with the same post-processing algorithm as shown in this study. However this is not a simple task as sequence development is rapid and therefore acquisition schemes are often updated and improved [30, 40–42]. Consequently, other approaches are desirable for normalization. Similarly, another problem regarding mapping results, that is not approachable by control of confounders, is the difference between field strength [7]. A proposed method to overcome this is the Z-score [16]. In this approach, mapping values are post-processed according to the standard deviation so that normalized values are obtained. The authors report that mapping values were comparable after this approach [16]. Another approach to standardize values across sequences, scanners and field strengths might be a clustering of acquired values and a comparison to published normative values [43]. Future approaches, however, should also focus on integrating technological, physiological and methodological confounders to provide comparable parametric results.

### Controllable and non-controllable confounders

Although the detected differences between sites I and III for the midventricular slice and segment 12 were statistically significant, the absolute difference (midventricular − 16.3 ms) should still be compared to the SD of this slice (± 34 ms for site I and ± 40 ms for site III). This illustrates the clinically neglectable relevance of this finding. However, controllable and non-controllable confounders which may have let to the differences between sites I/II and III, should briefly be touched upon. These include scanner version, bore size, surface coils, intrinsic physical tissue factors as well as operator experience. The scanner version of the same field strength seems to impact the results to a lesser extent as evidenced by the small differences between sites I and II, which run different scanner versions in comparison to sites II and III, which use the same scanner version. The same holds true for the bore size, which might have an effect on the results. The influence of coils as another potential confounder is difficult to assess as the sites I and III in comparison to site II used different coils however site I and III used a different scanner. One important confounder to note, however, might be the operator experience during image acquisition. This issue is unfortunately not only relevant to mapping but encompasses other aspects of CMR such as function, late gadolinium enhancement and flow assessment [44, 45]. We noticed a variable rate of analyzable segments across the sites with the highest rate being displayed at scanner site I. The scanner with the lowest rate, site III, also showed larger deviations compared to values at sites I and II (Fig. 3). Despite all influencing factors the septal segments, believed to be the most stable segments [46], showed no significant differences between the sites. Significant differences were found for the midventricular slice and on a segmental level for AHA segment 12. In the literature the lateral wall of the LV was described to have the most variable mapping values due to increased susceptibility artefacts (as confirmed in the present study), increased partial volume effect due to the heart–lung interference and is often more movable and thinner [21, 46, 47]. That led to the recommendation in the consensus statement to use septal regions [12].

With the question of interest of which of the confounding factors provided the intersite differences, this remains ultimately not answerable. More prospective data is needed to provide further insights into these findings. An introduction of prospective quality assurance as known from industry could be helpful in imaging labs as well. Nevertheless, in a retrospective setting the causes for unwanted deviations cannot be identified with certainty [48].

### Limitations

A limitation of the study is the relatively small number of healthy volunteers enrolled. Neither age nor BMI were representative. The effort of transporting and locally coordinating the scans, however, was challenging as every volunteer had to be scanned within a reasonable time period. No baseline data was available, therefore no statistical estimate of sample size could be made. The differences between site III and sites I and II are currently not fully explainable by this exploratory study, potentially signifying its relevance within a larger sample size. This warrants further studies including more diverse travelling volunteers. In addition to a small and young healthy cohort, no patients with cardiac disorders were included therefore limiting statements towards detection of abnormal values at the included sites.

## Conclusion

When confounders, such as field strength, intra-vendor diversity, acquisition schemes and post-processing analysis are controlled for, parametric mapping results are comparable between sites in multicenter studies.

### Supplementary Information

**Additional file 1.** Intra- and inter-reader comparisons.

## Data Availability

The datasets analyzed during the current study are not publicly available due to German laws but are available from the corresponding author on reasonable request.

## Abbreviations

CMR: Cardiovascular magnetic resonance
BER-CMR: Berlin Research Network for CMR
bSSFP: Balanced steady-state free precession
SAX: Short axis
LV: Left ventricle
cv: Chamber view
RV: Right ventricle
MOLLI: Modified Look-Locker inversion recovery sequence
FLASH: Fast low-angle shot sequence
SSFP: Steady-state precession
SD: Standard deviation
