# Supplementary figures and images for: Multi-site comparison of parametric T1 and T2 mapping: healthy travelling volunteers in the Berlin research network for cardiovascular magnetic resonance (BER-CMR)
Source: J Cardiovasc Magn Reson. 2023 Aug 14;25:47. doi: 10.1186/s12968-023-00954-9 (PMC10424349; doi:10.1186/s12968-023-00954-9)

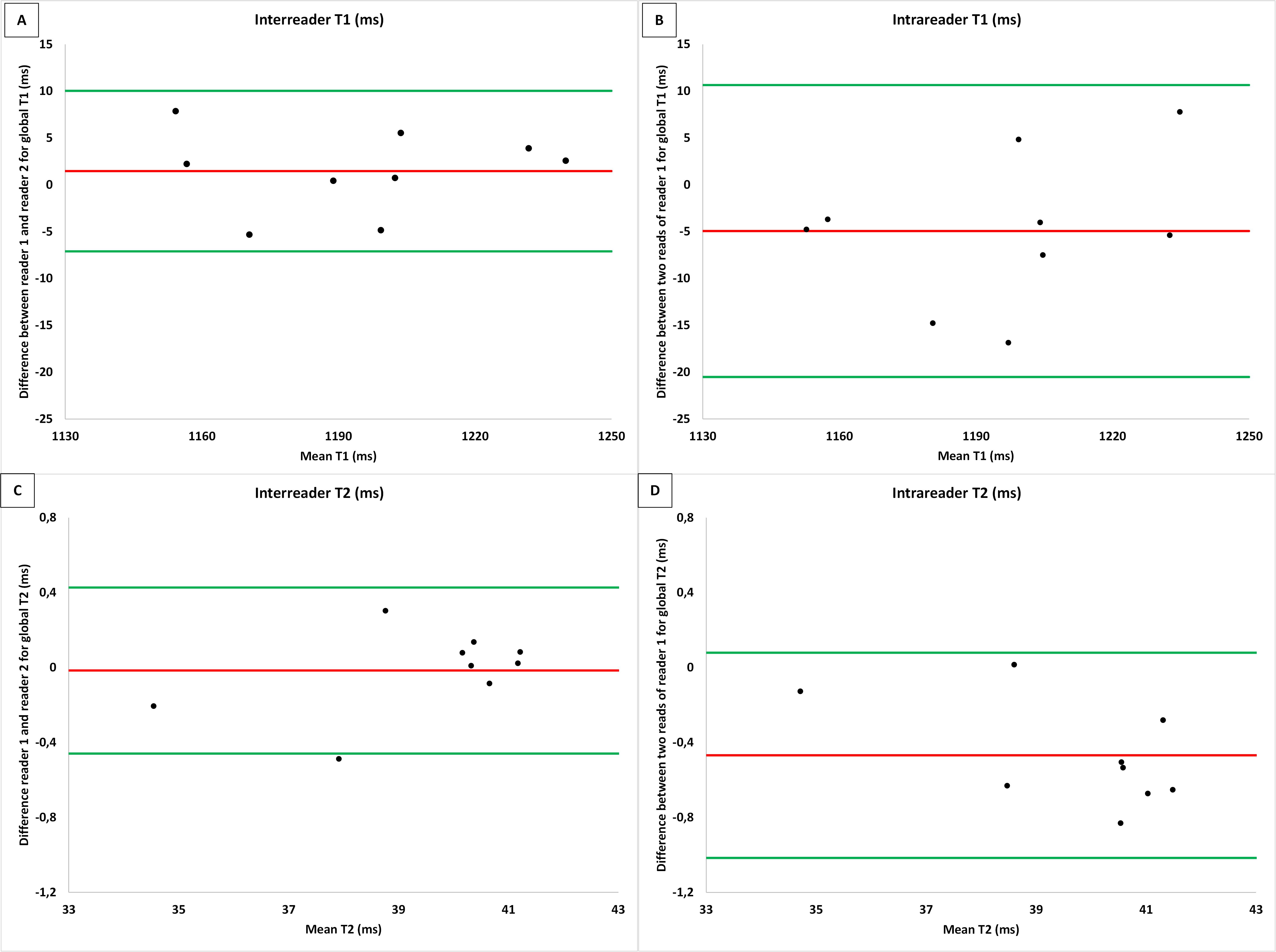

Supplement: Supplementary file 1 — Additional file 1. Intra- and inter-reader comparisons. [file 12968_2023_954_MOESM1_ESM.jpg]
